# Genetic Structure in Dwarf Bamboo (*Bashania fangiana*) Clonal Populations with Different Genet Ages

**DOI:** 10.1371/journal.pone.0078784

**Published:** 2013-11-14

**Authors:** Qing-qing Ma, Hui-xing Song, Shi-qiang Zhou, Wan-qin Yang, De-sheng Li, Jin-song Chen

**Affiliations:** 1 School of Landscape Architecture, Sichuan Agricultural University, Wenjiang, Sichuan, China; 2 China Conservation and Research Center for the Giant Panda, Wenchuan, Sichuan, China; 3 Institute of Ecological Forestry, Sichuan Agricultural University, Wenjiang, Sichuan, China; 4 College of Life Science, Sichuan Normal University, Chengdu, China; Universidade Federal do Rio Grande do Sul, Brazil

## Abstract

Amplified fragment length polymorphism (AFLP) fingerprints were used to reveal genotypic diversity of dwarf bamboo (*Bashania fangiana*) clonal populations with two different genet ages (≤30 years *versus* >70 years) at Wolong National Natural Reserve, Sichuan province, China. We generated AFLP fingerprints for 96 leaf samples, collected at 30 m intervals in the two populations, using ten selective primer pairs. A total of 92 genotypes were identified from the both populations. The mean proportion of distinguishable genotypes (G/N) was 0.9583 (0.9375 to 0.9792) and Simpson's index of diversity (D) was 0.9982 (0.9973 to 0.9991). So, two *B. fangiana* populations were multiclonal and highly diverse. The largest single clone may occur over a distance of about 30 m. Our results demonstrated that the genotypic diversity and genet density of *B. fangiana* clonal population did not change significantly (47 *versus* 45) with genet aging and low partitioned genetic differentiation was between the two populations (Gst = 0.0571). The analysis of molecular variance consistently showed that a large proportion of the genetic variation (87.79%) existed among the individuals within populations, whereas only 12.21% were found among populations. In addition, the high level of genotypic diversity in the two populations implies that the further works were needed to investigate the reasons for the poor seed set in *B. fangiana* after flowering.

## Introduction

Low recruitment rates from seeds have been reported to be common features in clonal populations even if seed production is abundant [1]–[4]. Proliferation of genetically identical individuals (ramets) in a population in which intraspecific competition occurs between both clones, and ramets within clones, potentially leads to few aggressive clones dominating the population [5]. For example, monoclonal populations have been found in *Populus tremuloides* (clone size of 81 ha, 10000-years old) [6] and *Pteridium aquilinum* (clone size up to 20 ha) [7]. This was thought to dramatically impoverish genotypic diversity in clonal populations. A prevailing assumption is that a high degree of asexual reproduction is often associated with genetic monomorphism [8], [9]. However, Ellstrand & Roose [10], Hamrick & Godt [11] and Widén *et al*. [12], in literature surveys of allozyme variation in clonal plants, concluded that clonal populations may have high genetic diversity. Such a high level of genotypic diversity has often been explained as a result of microsite heterogeneity that promotes the coexistence of clones through diversifying selection [10], [13]. However, the studies did not separate “asexual” species with frequent recruitment by sexually produced seeds from strictly asexual species with rare recruitment by sexually produced diaspores. The episodic sexual recruitment may be sufficient to compensate for mortality and maintain a high level of genotypic diversity in the clonal population [14].

Dwarf bamboo *Bashania fangiana* is a perennial monocarpic species, which has both types of sympodial and monopodial rhizome systems in a genet. It grows up to 2.0 m in height, vigorously extend rhizomes, and often form dense populations in the mixed coniferous and broad-leaved and subalpine coniferous forest understoreys, north-western Sichuan province, China [15]. *B. fangiana* flowers once about 50–60 years or even more seldom [15]–[17]. *B. fangiana* often flowers so simultaneously over an extensive area and then dies. After mass death, its populations recover mainly by the development of seedling cohorts. When the seedlings reach full size stage, they extend rhizomes and produce culms, resulting in dense populations again [18]. *B. fangiana* is one of the staple food bamboos for the giant panda (*Ailuropoda melanoleuca*), which is crucial to maintain its survival and reproduction [17], [19]. The bamboo is a monocarpic plant, which can only be reproduction by asexual after establishment, is an ideal experimental material to illustrate the levels of genetic diversity among clonal populations [20], [21]. No studies have been carried out to elucidate genetic structure within a *B. fangiana* population, and no information such as number of clones in a population is available. Because rhizomes of *B. fangiana* grow leptomorphically, different genets are likely to mix with each other, but no genetic identification of culms of *B. fangiana* stands has been done.

Amplified fragment length polymorphism (AFLP) [22], a more robust method that allows detecting many markers with high reliability, is now available and has been used in several studies for clonal structure of bamboo species [23]–[25]. In this paper, we have used the AFLP technique to identify individuals of *B. fangiana* in two 3.15 ha plots and to reveal the genetic structure of two *B. fangiana* populations with different genet ages (≤30 years *versus* >70 years). We addressed the following questions: (1) how many clones (genets) exist in two populations respectively; and (2) how large an area does a single clone occupy. In the complete absence of seedling recruitment, genotypic diversity (clonal diversity) is constrained to the level of the initial cohort and can only decline over time [26]–[29]. This inspires another problem: (3) if genotypic diversity of *B. fangiana* population will decline over genet age.

## Materials and Methods

### Ethics Statement

This study was carried out at an open field (102°51′–103°24′E, 30°45′–31°25′N) in Wolong National Natural Reserve, which is located in the southern slope of the Qionglai Mountains, Sichuan province, China. We obtained appropriate permissions from the Forestry Bureau of Wenchuan County, and assistance from the forestry workers for field study. In present study, *B. fangiana*, one of the staple food bamboos for the giant panda (*Ailuropoda melanoleuca*) and often forming dense populations in the mixed coniferous and broad-leaved and subalpine coniferous forest understoreys in natural reserve was used as investigated subject, and we confirmed that our studies did not involve endangered or protected species. In addition, no specific permission was required for these locations because our study was the general sampling experiment.

### Study site and sampling procedures

The aim of the Wolong National Natural Reserve is to conserve giant pandas and their habitat. It is in the subtropical area with an average annual temperature 8.42°C; −1.34°C in January and 17.06°C in July. Precipitation averages 884.24 mm year^−1^ with 76.66% of annual rainfall occurring between May and September. There are different types of vegatation with a vertical distribution: evergreen broad-leaved forest below 1600 m; mixed evergreen broad-leaved and deciduous broad-leaved forest between 1600 and 2000 m; deciduous broad-leaved forest between 1500 and 2600 m; mixed coniferous and broad-leaved forest between 2000–2600 m; subalpine coniferous forest between 2600 and 3800 m; and interspersed shrublands and meadows above 3800 m. *B. fangiana*, *Fargesia robusta* and *F. nitida* are the main panda grazing species within pandas' habitat in Wolong National Natural Reserve [15].

In september 2012, two rectangular sampling plots (150 m×210 m) were chosen near the Wuyipeng Research Station in Wolong National Natural Reserve. The two plots belonging to mixed coniferous and broad-leaved forest, were dominated by *Tsuga chinensis, Betula albo*-*sinensis*. *B. fangiana* forms dense population in the forest understorey. So, the light, soil and topographic conditions *et al* seemed to be uniform in the research plots. One plot (N1) was established after the mass-flowered of *B. fangiana* in 1983 and its genet age was not more than 30 years old. The other (N2) was at least more than 70 years old (Zhoushi Qiang, research expert from Wolong National Natural Reserve, personal communication). Within each plot, 48 leaf samples were collected at each intersection point on a 30×30 m grid and immediatly preserved in silica gel to prevent DNA degradation.

### DNA extraction and AFLP procedure

Total genomic DNA was extracted from young leaves using the Plant Genomic DNA kit (Tiangen Biotech Co. Ltd. Beijing) according to the manufacturer's instructions. Then, DNA samples were suspended in TE buffer. AFLP analysis consists of four steps: restriction digestion, adaptor ligation, preselective amplification and selective amplification and was conducted essentially as described by Vos et al. [22]. The ten selective pairs of *Mse*I(M-) and *Eco*RI(E-) primers used in the selective amplification were identified as the most informative and reliable from a set of 256 primer pairs during a preliminary investigation (Table 1). The amplified AFLP loci were run in a 48-well comb, which allowed processing of one population at the same time. After electrophoretic separation of the amplification products on polyacrylamide sequencing gels, the different AFLP loci were visualized by sliver staining and scored manually against a known nucleotide sequence used as a molecular-size marker. We classified those fragments to 1 (presence) or 0 (absence) in a readable range between 100 and 1500 bp to generated AFLP binary matrices (Table S1), and the data matrix of the AFLP phenotypes was constructed for further analysis.

**Table 1:** List of 10 pairs of screened primers.

No.	EcoR I Primer	Mse I Primer
1	5′-GACTGCGTACCAATTCAAT-3′/(E-AAT)	5′-GATGAGTCCTGAGTAACAC-3′/(M-CAC)
2	5′-GACTGCGTACCAATTCACA-3′/(E-ACA)	5′-GATGAGTCCTGAGTAACTC-3′/(M-CTC)
3	5′-GACTGCGTACCAATTCACT-3′/(E-ACT)	5′-GATGAGTCCTGAGTAACGT-3′/(M-CGT)
4	5′-GACTGCGTACCAATTCACC-3′/(E-ACC)	5′-GATGAGTCCTGAGTAACGA-3′/(M-CGA)
5	5′-GACTGCGTACCAATTCACC-3′/(E-ACC)	5′-GATGAGTCCTGAGTAACGT-3′/(M-CGT)
6	5′-GACTGCGTACCAATTCACG-3′/(E-ACG)	5′-GATGAGTCCTGAGTAACGT-3′/(M-CGT)
7	5′-GACTGCGTACCAATTCATG-3′/(E-ATG)	5′-GATGAGTCCTGAGTAACGT-3′/(M-CGT)
8	5′-GACTGCGTACCAATTCATC-3′/(E-ATC)	5′-GATGAGTCCTGAGTAACGC-3′/(M-CGC)
9	5′-GACTGCGTACCAATTCATA-3′/(E-ATA)	5′-GATGAGTCCTGAGTAACGT-3′/(M-CGT)
10	5′-GACTGCGTACCAATTCATA-3′/(E-ATA)	5′-GATGAGTCCTGAGTAACGC-3′/(M-CGC)

### Data analysis

Samples showing the same multilocus AFLP phenotype were considered to be the same genotype. To determine the genotypic diversity (clonal diverdity) in the two populations with different genet ages, three measures were calculated following Ellstrand & Roose [10]: (i) the proportion of distinguishable genotypes (G/N), where G is the number of genotypes and N is the sample size; (ii) Simpson's index of diversity corrected for finite sample size [30], given by D = 1−{[∑N_i_(N_i_-1)]/[N(N-1)]}, where N_i_ is the number of samples with genotype i and N is the total number of samples; and (iii) genotypic evenness [31], calculated as E = (D-D_min_)/(D_max_-D_min_), Where D_min_ = [(G-1)(2N-G)]/[N(N-1)] and D_max_ = [N(G-1)]/[G(N-1)]. *D* ranges 0 in a population composed of a single clone to 1 in a population where every sampled individual represents a different clone. *E* ranges from 0 in a population where all samples represent different clones, or where one clone dominates and the other clones are represented by a single sample, to 1 in a population where all clones are represented by the same number of samples. All three measures of genotypic diversity were based on the 96 samples collected at each intersection point on the 30×30 m grid in the two populations.

We also used POPGENE 1.31 to calculate four genetic diversity indices within each population, that are commonly used in the literature: the number of polymorphic loci, the percentage of polymorphic loci (PPB), the Nei's [32] gene diversity (h) and Shannon's information index(I). Using the same program, the coefficient of gene differentiation (Gst) was estimated, and gene flow (Nm) among populations was also indirectly estimated by a traditional method based on Gst (Nm = 0.5(1-Gst)/Gst). An analysis of molecular variance (AMOVA) using AMOVA(ver 1.55) was performed to describe the genetic structure and variation within the populations and among the populations. To visualize the genetic relationships among the populations, Dice similarity coefficient was calculated to develop a dendrogram based on the unweighted pair group method of cluster analysis that used arithmetic averages (UPGMA). The computer package NTSYS-PC (Numerical Taxonomy and Multivariate Analysis System) Version 2.10e was used for cluster analysis. Principal component analysis (PCA) was also carried out to check the results of UPGMA-based clustering using the EIGEN module of NTSYS-PC.

## Results

We obtained AFLP binary matrices from 96 *B. fangiana* ramets samples. The observed numbers of bands by AFLP analysis were 202 using the ten selective primer pairs, each primer pair produced an average of 20.2 distinct bands. Excluding commom bands, ten primer pairs yielded a total of 111 polymorphic bands among these 96 samples. 107 were polymorphic in the N1 ramet population, and 91 were polymorphic in the N2 ramet population (Table 2).

**Table 2:** Genetic diversities measured as number of polymorphic loci, percentage of polymorphic loci(PPB), nei's gene diversity (h) and Shannon's information index(I).

Population	Total number of loci	Number of polymorphic loci	Percentage of polymorphic loci(PPB)	Nei's gene diversity (h)	Shannon's information index(I)
N1	202	107	52.97%	0.1463(±0.1750)	0.2286(±0.2555)
N2	202	91	45.05%	0.1229(±0.1704)	0.1920(±0.2494)
Species	202	111	54.95%	0.1428(±0.1680)	0.2265(±0.2461)

**Figure 1:**
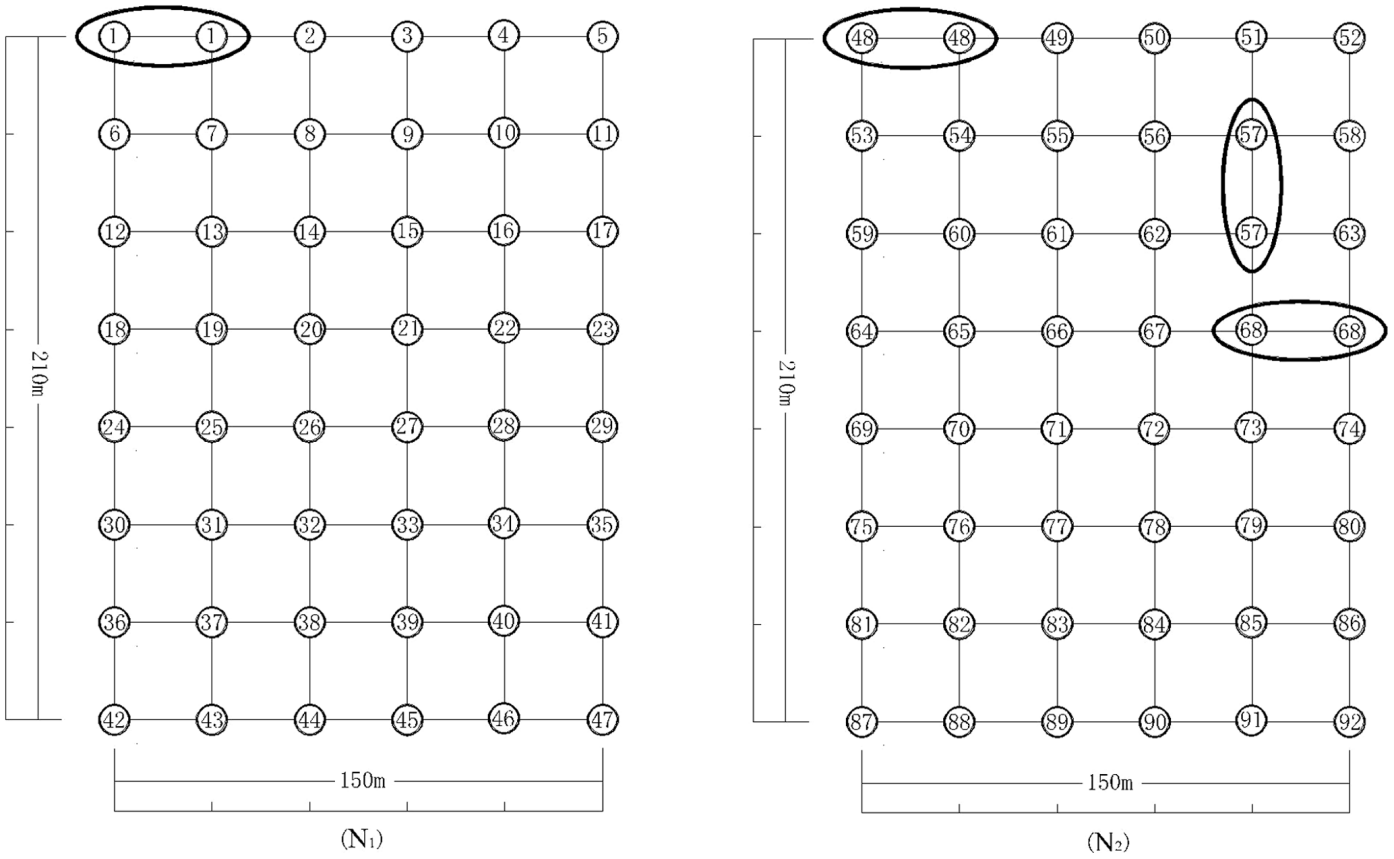
Clonal structure of the two populations of *B. fangiana* inferred from amplified fragment length polymorphism (AFLP) fingerprints in two 3.15 ha study plots. Numbers indicate the sampling ramets in two plots, the different numbers indicate the different genotype and the same numbers (in the ellipse) indicate the same genotype.

Among 96 *B. fangiana* samples, 92 genotypes (G = 92) with identical AFLP fingerprints were detected. The clonal structure of the two populations is depicted in Fig. 1. The number of genets in N1 and N2 populations was 47 and 45 respectively. No shared genotype was found in both populations. In the N1 population, the proportion of distinguishable genotypes (G/N) was 0.9792. Estimates of Simpson's index of diversity (D) and genotypic evenness (E) were 0.9991 and 0 respectively; The corresponding values for the N2 population were 0.9375 (G/N), 0.9973 (D) and 0.6667 (E). The mean proportion of distinguishable genotypes (G/N) and Simpson's index of diversity (D) for *B. fangiana* was 0.9583 and 0.9982, respectively (Table 3). The parameters of genetic diversity in the two populations calculated from polymorphic AFLP bands are listed in Table 2. At the species level, the percentage of polymorphic loci (PPB), Ni'se gene diversity (h) and Shannon's information index (I) were 54.95%, 0.1428(±0.1680), 0.2265(±0.2461), respectively. At the population level, the value of PPB per population were 52.97% and 45.05%. The Nei's gene diversity (h) and Shannon's information index(I) were h_N1_ = 0.1463(±0.1750) and h_N2_ = 0.1229(±0.1704); I_N1_ = 0.2286(±0.2555) and I_N2_ = 0.1920(±0.2494), respectively. So, there was no great difference in genotypic diversity and genetic diversity between N1 and N2 populations.

**Table 3:** Parameters of genotypic diversity for each ramet population.

Population	N	G	G/N	D	E
N1	48	47	0.9792	0.9991	0
N2	48	45	0.9375	0.9973	0.6667
Mean	48	46	0.9583	0.9982	0.3334

N, sample size; G, number of genets; G/N, proportion of distinguishable genotypes; D, Simpson's index of diversity and E, genotypic evenness.

The results from our analysis of molecular variance (AMOVA) showed that a large proportion of the genetic variation (87.79%) existed among individuals within the populations, and that 12.21% resided among the populations. The genetic differentiation among the populations was significant (P<0.001) (Table 4). Consistently, Nei's estimator of population genetic structure also indicated a low level of population differentiation (coefficient of gene differentiation, Gst = 0.0571). The estimate of gene flow (Nm) among populations was 8.2639.

**Table 4:** Analysis of molecular variance in two populations of *B. fangina*.

Source of variance	D.f.	Sum of Squares	Variance component	Percentage of variation(%)	P-value
Variance among population	1	113.375	2.0542	12.21	*P*<0.001
Variance within population	94	1388.7083	14.7735	87.79	*P*<0.001

**Figure 2:**
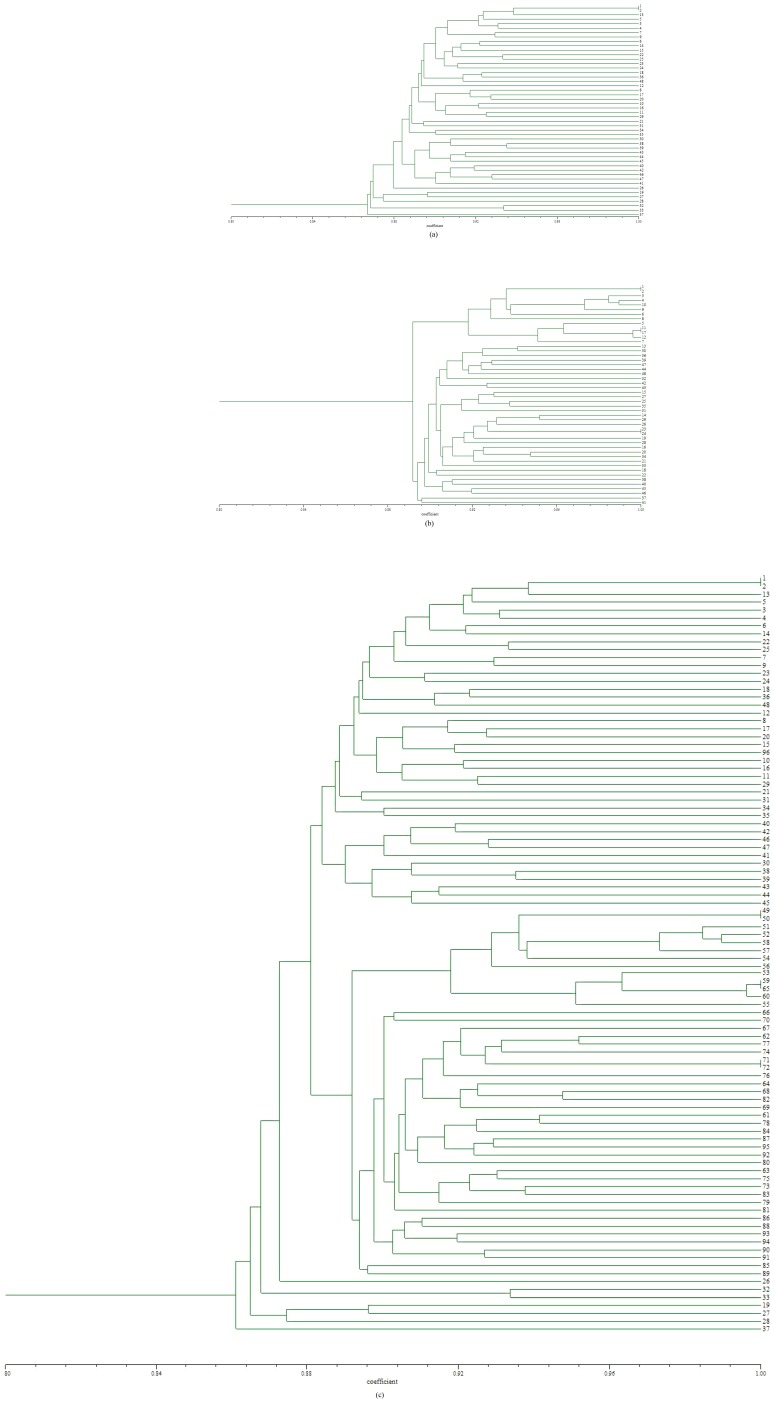
Dendrogram of UPGMA cluster analysis of *B. fangiana* N_1_ population (a), N_2_ population (b) and both the two populations (c). (In the UPGMA dendrogram of (c), 1 to 48 indicate the ramets of *B. fangiana* sampled from N_1_ population, and 49 to 96 indicate the ramets sampled from N_2_ population.)

**Figure 3:**
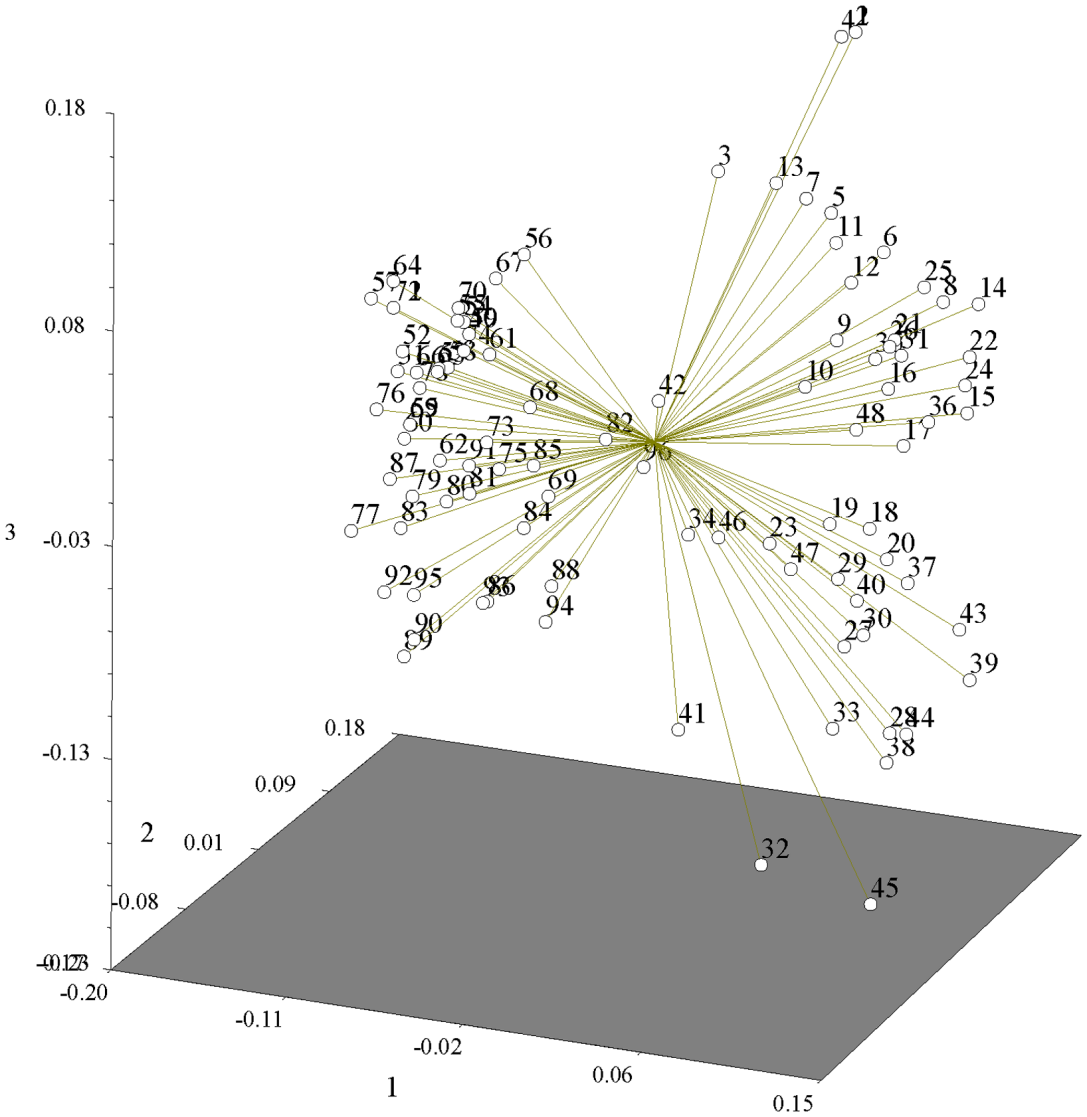
Three-dimensional plot of the 96 samples of *B. fangiana* based on principle component analysis (PCA).

The largest size of a single clone in our study area might cover a distance of about 30 m at least (Fig. 1). The UPGMA cluster analysis revealed two distinct groups (Fig. 2(c)): I, the 41 ramets sampled from population N1; II, the 48 ramets (number from 49 to 96) sampled from population N2. Ramets of the same *B. fangiana* population first clustered into one group, then clustered with other population. Only a small number of ramets (ramets 19, 26, 27, 28, 32, 33 and 37) did not gathered together with other ramets in the same population (N1), which suggesting larger genetic variation compared with others in the population. Results of the UPGMA analysis also revealed that the high genetic similarity were shared between the two populations (Fig. 2). In PCA based on the AFLP data matrix of the 202 bands for 96 ramets in two *B. fangiana* populations, the first three components effectively distinguished the two populations and ramets of the same population gathered close, which was consistent with the results of UPGMA (Fig. 3).

## Discussion

Many clonal plant populations usually can be as diverse as those of non-clonal plant species [33]–[35]. The average genotypic diversity and evenness values in *B. fangiana* in this study (G/N = 0.9583; D = 0.9982; E_N1_ = 0, E_N2_ = 0.6667) were higher than the average values found in other clonal plants (G/N = 0.17; D = 0.62; E = 0.68) [10]. Nevertheless, the high genotypic diversity found in *B. fangiana* is confirmed by the fact that both of the sampled populations were multiclonal, compared to an average of 62% in Ellstrand and Roose [10] and Widén et al. [12]. Our results also showed that the *B. fangiana* species had the levels of percentage of polymorphic loci (P = 54.95%) and Nei's gene diversity (h = 0.1428) similar to the average values calculated for populations of 120 wind pollinated outcrossing taxa (P = 53.0%; h = 0.154) [36]. Maintenance of genotyipc diversity in clonal population is determined by the ratio of genet extinction to the recruitment of new genets [27]. In many clonal species, seedling recruitment has only been found to occur in short colonization episodes after large-scale disturbances [1]. If no further recruitment from seedling occurs, ramet density of clonal plant populations may become so large that self-thinning can be expected [37]. Such a recruitment strategy has been referred to as Initial Seedling Recruitment (ISR) [1], [38], [39], with which the genotypic diversity of a clonal population can only decline over time as a result of the competitive exclusion of genotypes or stochastic mortality [5], [40]. *B. fangiana* is monocarpic, often flower simultaneously and die subsequently over intervals of several decades [41], and then regenerating from seed. It is suggested that *B. fangiana* is more like a typical ISR species in the ISR-RSR spectrum of Eriksson.

The genetic and genotypic diversity could be influenced by a number of processes (e.g., mode of reproduction, gene flow, mutation, selection) [42]. The high genotypic diversity in *Sasa senanensis* were incurred by the environmental heterogeneity [23], [43]. Selection for particular ecotypes in different environment of a species range would contribute to variation in diversity among populations [44]. However, the two plots were homogeneous in the study. In addition, the genotypic diversity was high and did not decrease over time. The environmental homogeneity for the two plots implies that local adaptation may not be the factor resulting in the current genetic structure. Some studies that followed genotypic diversity through time with quantitative markers supported the theoretical prediction of a decrease in genotypic diversity with time [29], [45]; However, no decrease in genotypic diversity over time was observed [27], [42], [46], [47]. In *B. fangiana* with a obligatory xenogamy breeding system, the genotypic diversity of its seedlings establishing a new population is expected to be high compared with other less out-crossed species. So, the maintenance of genetic diversity through time could be fostered by the particular reproductive system of *B. fangiana*. Bengtsson [48] proposed that clonal populations possess an effective “memory” of their earlier genetic history, in the way that “a population which was started by a number of sexually derived propagules may thus retain its initial genotypic variation for a very long period of time, even if it later reproduces almost exclusively asexually”. An initial burst of sexual recruitment when the population was founded after mass-flowered could compensate for the loss of diversity within a population. *B. fangiana* can only reproduction by asexual after established. Therefore, founder effects in *B. fangiana* might be an important mechanism for maintaining in a high level of neutral genetic variability.

The alternative explanation is that the high level of genetic diversity is also maintaining through clonal growth. Clonal growth could retard the loss of genetic diversity within populations by several mechanisms [12]. Independent ramets could spread the risk of mortality among ramets [49]–[51], thus reducing the probability of genet death and hence preserving genetic variation.

In addition, the higher genetic diversity in our study might result from the higher power of resolution of the DNA-based molecular marker (AFLP), and sampling scheme (e.g. the scale and the size of mesh) with which ramets are sampled also influences these estimates of genotypic diversity.

Small genetic differentiation was detected in *B. fangiana* by low Gst value 0.0571, which was supported by the AMOVA analysis that partitioned genetic variation among populations was only 12.21%. Many factors can determine the genetic structure of plant populations, including the reproductive biology [52], and gene flow [53]. Wright [54] argued that clear genetic structure would arise when inbreeding occurred, whereas outcrossing species tended to show little genetic structure. Many RAPD- and sequence tagged microsatellite sites (STMS)- based analyses showed that long-lived and outcrossing taxa retained most of their genetic variability within populations [55]. As one of those critical influences, the outcrossing of a plant species tends to explain 10 to 20% of the genetic variation among populations, whereas the selfing of a species leads, on average, to 50% variation between populations [11]. The woody bamboos have a long vegetative phase of 20–150 years, and are typical long-lived species of the grass family. Therefore, the low level of genetic differentiation among *B. fangiana* populations might result from its extensive longevity, obligate xenogamy reproductive system and the large gene flow (Nm = 8.2639). UPGMA dendrogram and PCA analysis further exhibited that low partitioned genetic variation existed between the two populations which implied the high genetic similarity were shared between the two populations. This outcome could have reflected the possibility that the examined populations of *B. fangiana* still represented the abundant genetic variation of their ancestors which was supported by the high level of genetic diversity in the two populations. In fact, this tendency is reinforced by the naturally long vegetative phase and low seed set over the life cycle, which are typical features of woody bamboos [56]–[58]. Additionally, population genetic structure is highly influenced by the environment [43]. The highly uniform environment in the two populations may be one of the factors resulting in the current genetic similarity.

*B. fangiana* populations are not dominated by one clone or have any dominate genotype and we did not observe any mixed distribution of multiple clones. The largest size of a single clone might occurs a distance of about 30 m at least, which was consistent with a excavation study showing that the annual rhizome increments of *B. fangiana* was about 45 cm [15].

*B. fangiana* may be an obligate xenogamy plant [15]. A 6-year study showed that although the pollen viability of *B. fangiana* was very high, but the seed setting rate was rare after flowering [15]. Our results clarify, for the first time, the poor seed set in *B. fangiana* may not caused by the low level of genotypic diversity and other reason may need to be responsible for it. The pistil and stamen are not mature at the same time or the disadvantage of its own physical/physiological structure and morphology may contribute to the seed sterility [59]. To determine whether our results can be extrapolated to other populations and to reveal the real reason that lead to the poor seed set in *B. fangiana*, the further study may be needed.

## Supporting Information

Table S1 **Binary matrix of AFLPs result for empirical AFLP analysis.**
(XLS)
